# New Drugs, Appliances, and Things Medical

**Published:** 1891-02-28

**Authors:** 


### NEW DRUGS, APPLIANCES, AND THINGS MEDICAL.

### "CORALLINE" FOOD.

Messrs. George Rogers, Gerds, and Co., Leadenhall Street, have asked us to test the above and report on the same. It is a maize food prepared in a special way, so that further extensive cooking is unnecessary; but this preparation has not "torrified" the flour, thus chemically altering its constitution. The maize is the finest selected southern growth, which is produced entirely for human consumption, and there are none of the commoner varieties of Indian corn used in the manufacture of Coralline. The manufactured material contains all the grain except its hull. The flour is steamed and dried at a high temperature, but not so high as to produce the above-mentioned alteration. It is contended by the manufacturers that all the nutritious parts of maize are contained in Coralline. Our examination leads us to think very favourably of this food. It is pleasant to the eye, and when tasted raw has that clean nutty flavour which no process of manufacture can give to an inferior cereal food. The usual chemical tests demonstrate its freedom from impurity, and when cooked, it produces an appetising dish which is a decidedly pleasant variety in the way of milk puddings. It forms a useful food for children, invalids, and the dyspeptic.

